# Worcester Infirmary

**Published:** 1830-01-01

**Authors:** 


### WORCESTER INFIRMARY.

I. Empyema, with External Tumour.

Th. Booth, æt. 62, was admitted into the Worcester Infirmary, April 5th, 1828, under the care of Mr. Sheppard, with an oblong tumour, about the size of the section of a turkey's egg, situated over the cartilages of the four inferior ribs on the right side, about four inches below the mamma. It was free from pain; of irregular form, with a disk of inflammation on three of its most prominent points; firmly attached and immoveable at its base; lobulated and knotty in its feel; somewhat elastic upon pressure; and giving the sensation of a fluid in different cysts. He said that he had struck the part in September, that the pain, which was first severe, soon abated, and that two months after the injury he discovered the tumour then about the size of a large nut. Since that time it had gone on increasing, but he attributed the inflammation on the surface, to the use of a strong solution of salt, about three weeks before his admission.

On the 8th, the tumour was punctured with a lancet, when there issued a quantity of fetid pus, containing an innumerable quantity of hydatids, most of them about the size of small nuts. The integuments collapsed, but on coughing, the tumour instantly filled, and pus and hydatids spouted from the wound, when the effort was made. A small dossil of lint was inserted into the wound, a poultice laid over it and the patient sent to bed. In the night of the 9th, he had rigors, and on the 10th there was fever, urgent cough with dyspnœa, and pain about the wound with abundant discharge. Leeches and purging procured relief, and on the 12th the former were repeated, with a bolus of five grains of calomel and five of antimonial powder, followed by castor oil, and salines with antimony. On applying the stethoscope above the right nipple, the respiratory murmur was very distinct, whilst below that part it could not be heard. On directing him to cough the gurgling of fluid was heard in the chest; the left side appeared to be sound on auscultation.

We need scarcely pursue the diurnal details, suffice it that the cough and expectoration lessened, on the 26th, the respiratory murmur was heard below the right mamma, and on the 28th of June he left the infirmary. He was at this time much improved in his general health, the cough was very slight, and the discharge reduced to the quantity of an oz. and a half in the 24 hours. He returned to a laborious employment, that of a woodman, and presented himself at the Infirmary in last June, apparently in very good health, having merely a little discharge from the wound occasionally.

We think it must be evident that the above was not a case of empyema, in the fair acceptation of the term, that is, a case in which pus was effused into the general cavity of the pleura. The gradual progress of the complaint, the little comparative disturbance to the respiratory system, the limited tumour, the character of the discharge, the auscultic indications, and last not least the very successful issue of the case, all combine to prove that the pus and hydatids were confined in a restricted cavity, most feesably between the pleuræ, with surrounding adhesions of those membranes. This encysting of fluid by partial adhesions of the pleuræ plays a very important part in modifying symptoms, whether that fluid be serum or pus. In the latter case, or encysted empyema, if we may use such a term, the patient has a thousand times better chance of ultimate recovery from an operation, than when the matter roams abroad in the general pleural cavity. We could give some interesting cases to illustrate these points were space allowed, but for the present we must content ourselves with merely hinting thus briefly at the subject.

### II. HEMIPLEGIA—EMPLOYMENT OF STRICHNINE.

"James Jevons, æt. 10, came into the Infirmary, May 30th. Has partial paralysis of the right side; occasional headache; the pupil of the left eye contracts very irregularly; pain and tenderness in the hypogastrium; intellect much impaired; memory very bad; looks idiotic; tongue, when projected from the mouth, is directed towards the paralytic side; bowels costive; tongue clean; pulse 84, weak. About Christmas last, had a fall from a cart, and received a severe wound over the left orbit, from which time he has complained of occasional head-ache. Has had symptoms of hemiplegia for five weeks; has been under surgical care, but obtained only temporary relief. Applic. hirud. xii. lateri capitis sinist. Sumt. haust. cathart. 1oz. statim. et repet. post horas tres, si opus fuerit.

"June 2nd.—Is much relieved by the application of the leeches; head more free from pain; can raise his arm with more ease; walks better; pupil of the left eye contracts more regularly; tongue projected from the mouth in a straighter line, can move it to the left side with ease, which he could not do before; answers questions more readily. 3rd.—Rept. hirud. cras; perstet in usu haustus cathart. omni mane. 5th.—Is much better since the application of the leeches. 7th.—Applic. emp. lyttæ lateri capitis sinist. postea. Ung. antim. tart. ibidem. 9th.—Can use his extremities with much more facility; pupil of the left eye contracts naturally. 14th.—Sumt. mistur. cathart. p. r. n. 16th.—Opens the hand much easier, the extremities much less paralytic. 19th.—Sumt. strichninæ, gr. 1-6 ter indies. 24th.—Continues to improve. Rept. emp. lyttæ capiti.

"July 7th.—Has continued to improve under the use of the strichnine; augeatur dosis strichninæ ad gr. 1-3 ter die."

As improvement evidently *preceded* the strichnine in the present case, and as the drug was only exhibited from the 19th of June to the 7th of July, (for there the report stops,) we cannot in justice attribute very much to its influence. However, we are witnessing a case of paraplegia in a boy, where the strichnine would seem to be producing good effects, and no doubt can exist that the remedy has often proved of service. It is worth experimenting with in these obstinate and melancholy cases.

III. SUPPOSED HEPATITIS.

Ann Williams, *twenty* years of age, was admitted on the 30th of May, with severe pain and tenderness in the hepatic region and abdomen—slight pain in the *head*—catamenia regular but *profuse, and occasioning great debility during their continuance*—pulse 84 and *weak*—appetite bad—want of sleep at night—tongue white and loaded—bowels open. She had been ill five days, and attributed the attack to cold from going out of doors insufficiently clad. Previous to this, she had had a cold in her head to which she was very subject, and fourteen months before, she had been laid up for four months with a similar attack to the present, preceded by an inflammation in the left elbow. Twelve leeches were applied to the right side, with relief to the pain, and she was ordered next day an antimonial pill and sulphate of magnesia. On the 1st of June she was better, but complained of pain in the right side of the chest. On the 5th, the pain in the side was increased and leeches were again applied. On the 7th, the pain in the side was better, and the pain in the head was gone. On the 9th, there was a slight relapse of the former, the leeches were repeated, and on the 14th, she was able to be made out-patient.

We really see not a shadow of evidence that *hepatitis* existed in the foregoing case, unless *pain* in the *right* side be considered a sufficient pathognomonic sign of the disease. In point of fact, this case was one of those nervous or hysterical cases which are constantly met with in practice, and constantly mistaken and mistreated. A *young* woman with disordered menstruation, in some cases it is scanty in others profuse, is attacked with severe pain in the right side or the left, perhaps with a little cough, almost always accompanied with pain in the head. A routine or unwary practitioner will take the alarm, inflammation will be conjured up, and the long array of depleting measures be directed against the supposed phlogosis, till the patient is either strong enough to throw off the disease, and what is ten times worse the doctor, or sinks into a state of hopeless debility. Unfortunately this is not an imaginary case, and at the present moment we have before our eyes a melancholy instance of such mischievous medication. With proper precautions, these hysterical pains are not difficult to distinguish from those that depend on inflammation or organic alterations. They are always too general for the latter, and exist at the same time, or in quick alternation, in the head, the chest, the liver, the colon;—they are mostly superficial and aggravated to an extraordinary degree by the *slightest* pressure; they are much more severe than the sufferings from actual disease, which, in fact, they caricature; and finally, they are exasperated in the long run, by depletion, at least in ninety-nine cases out of the hundred. As we previously observed, they occur for the most part in young unmarried females, with disordered menstruation, and very frequently indeed in those affected with chlorosis. The general health is often good, occasionally indifferent, but *never* implicated in proportion to the local sufferings. There is commonly costiveness of the bowels, and much flatulence; not seldom the *globus*, and other decidedly hysterical symptoms.

These complaints are annoying and obstinate at the best, but under improper treatment their inveteracy and intensity are increased a hundred fold. Depletion will never do; on the contrary, those remedies are indicated that regulate the bowels and the menstrual secretion, and improve the general health. The practitioner must choose his remedies from the list of antispamodics, emmenagogues, and tonics, not from the deadly catalogue of antiphlogistics. The subject is one of such practical importance, that we shall return to it more at large on a future occasion.

